# The Combination of Phages and Faecal Microbiota Transplantation Can Effectively Treat Mouse Colitis Caused by *Salmonella enterica* Serovar Typhimurium

**DOI:** 10.3389/fmicb.2022.944495

**Published:** 2022-07-07

**Authors:** Xinwu Wang, Yating Xing, Yalu Ji, Hengyu Xi, Xiaohe Liu, Li Yang, Liancheng Lei, Wenyu Han, Jingmin Gu

**Affiliations:** ^1^State Key Laboratory for Zoonotic Diseases, Key Laboratory of Zoonosis Research, Ministry of Education, College of Veterinary Medicine, Jilin University, Jilin, China; ^2^The Second Hospital of Jilin University, Changchun, China; ^3^Jiangsu Co-innovation Center for the Prevention and Control of Important Animal Infectious Diseases and Zoonoses, Yangzhou University, Yangzhou, China

**Keywords:** *Salmonella enterica* serovar Typhimurium, colitis, phage cocktail, FMT, SCFA

## Abstract

*Salmonella enterica* serovar Typhimurium (*S.* Typhimurium) is one of the common causes of human colitis. In the present study, two lytic phages vB_SenS-EnJE1 and vB_SenS-EnJE6 were isolated and the therapeutic effect of the combination of phages and faecal microbiota transplantation (FMT) on *S.* Typhimurium-induced mouse colitis was investigated. The characteristics and genome analysis indicated that they are suitable phages for phage therapy. Results showed that vB_SenS-EnJE1 lysis 41/54 *Salmonella* strains of serotype O4, and vB_SenS-EnJE6 lysis 46/54 *Salmonella* strains of serotypes O4 and O9. Severe inflammatory symptoms and disruption of the intestinal barrier were observed in *S.* Typhimurium -induced colitis. Interestingly, compared with a single phage cocktail (Pc) or single FMT, the combination of Pc and FMT (PcFMT) completely removed *S.* Typhimurium after 72 h of treatment, and significantly improved pathological damage and restored the intestinal barrier. Furthermore, PcFMT effectively restored the intestinal microbial diversity, especially for Firmicutes/Bacteroidetes [predominantly bacterial phyla responsible for the production of short-chain fatty acids (SCFA)]. Additionally, we found that PcFMT treatment significantly increased the levels of SCFA. All these data indicated that the combination of phages and FMT possesses excellent therapeutic effects on *S.* Typhimurium -induced intestinal microbiota disorder diseases. Pc and FMT played roles in “eliminating pathogens” and “strengthening vital qi,” respectively. This study provides a new idea for the treatment of intestinal microbiota disorder diseases caused by specific bacterial infections.

## Introduction

*Salmonella enterica* serovar Typhimurium (*S.* Typhimurium) is one of most important pathogens causing enterocolitis in humans (Collaborators, 2017), which is characterised by the increased colonisation of *S.* Typhimurium in the intestine and the strong inflammatory response leading to colitis (Barthel et al., 2003). This foodborne pathogen causes a significant public health threat and considerable economic burden worldwide (Majowicz et al., 2010; Jajere, 2019).

Bacteriophage (phage) is a promising biocontrol strategy due to the high specificity to given bacterial species, and it thus shows minimal side effects to normal microbiota (Abedon, 2011; Alomari et al., 2021; Hassan et al., 2021). Several reports have shown the successful applications of phage therapy in animal models against different bacterial infections, such as *Pseudomonas*, *Escherichia coli*, *Salmonella*, and *Enterococcus faecalis* (Khan Mirzaei and Nilsson, 2015; Cheng et al., 2017; Waters et al., 2017; Tang et al., 2019). However, the emergence of phage-resistant variants has also frequently been reported in several animal models as well as in human pilot studies and case reports (Oechslin, 2018; McCallin et al., 2019). Phage cocktail could circumvent bacterial resistance more effectively than a single phage (McCallin et al., 2019; Pires et al., 2020). However, there are some studies in which bacteria cannot be completely removed even after the application of phage cocktail (Oliveira et al., 2010; Rivas et al., 2010). Additionally, most reports using antibiotics against *Salmonella* infections have led to intestinal microbiota disorders (Baumler and Sperandio, 2016) and a marked expansion of bacteria in the lumen of the murine intestine (Rivera-Chávez et al., 2016). However, phage treatment has no beneficial effect on the recovery of the intestinal microbiota. Therefore, phages combined with other therapeutic strategies that can restore the intestinal microbiota are being developed.

It has been reported that faecal microbiota transplantation (FMT) consists of the administration of faecal material into the recipient’s intestine to regulate the microbiota disorders and restore health. It has been used to treat recurrent *Clostridium difficile* infection for many years with a cure rate of 85–90% in patients (Van Nood et al., 2013; Lahtinen et al., 2017). Recently, it has been successfully used to alleviate mastitis caused by *Staphylococcus aureus* infection (Hu et al., 2020). In addition, FMT has shown potential for the intestinal decolonisation of extensively antimicrobial-resistant opportunistic pathogens, such as vancomycin-resistant *Enterococci* (Singh et al., 2014; Manges et al., 2016; Davido et al., 2017). However, even a balanced gut microbiota can only remove a small number of pathogens without specific bactericidal effects (Borewicz et al., 2015). Therefore, combining phage therapy and FMT may not only kills pathogens specifically, but also regulates the intestinal microbiota and removes pathogenic residues that phages fail to remove.

In this study, FMT was combined with phage cocktail to treat *S.* Typhimurium-induced colitis in mice. We found that the combination therapy completely removed *S.* Typhimurium and effectively reversed the symptoms of colitis, which was superior to single phages or single FMT treatment. Analysis of intestinal microbiota indicated that the combination of phage cocktail with FMT restored the intestinal microbial diversity and the short-chain fatty acids (SCFA) levels. In this combination therapy, phage cocktail and FMT played roles in “eliminating pathogens” and “strengthening vital qi,” respectively.

## Materials and Methods

### Animals

Female BALB/c mice (weights of 18–20 g) at 6–8 weeks old were purchased from Changchun Yisi Experimental Animals, Changchun, China. All of them were normally kept for a week to adapt to new surroundings. SPF grade mice breeding fodder and water were supplied *ad libitum*.

### Bacterial Strains and Culture Conditions

The strains used in this study are listed in Supplementary Table 1. *Salmonella* strains were identified by universal primers (F: 5′-GTGGCGGACGGGTGAGTAA-3′, R: 3′-GTGTGACCTTGACTTCGTGCC-5′), serotyped by *Salmonella* antiserum (Ningbo Tianrun Biological Pharmaceutical Co., Ltd., Zhejiang, China), and cultured in Lysogeny broth (LB) (Becton, Dickinson and Company, United States) at 37°C shaker or on *Salmonella-Shigella* (*SS*) selective agar (Hopebio, China) at 37°C incubator. *E. coli* and *Klebsiella pneumoniae* strains were cultured in LB broth at 37°C shaker. *S. aureus* and *E. faecalis* were cultured in brain heart infusion (BHI) broth (Becton, Dickinson and Company, United States) at 37°C shaker.

### Phage Isolation and Characteristics

*Salmonella typhimurium* 01E (NCBI no. SRR17717446) (Wang et al., 2019) was used as a host to isolate phages from sewage samples collected from the Changchun sewage system and yak faecal samples collected from Tibet, China (Gong et al., 2016). Yak faecal samples were soaked overnight in 10 times the volume of SM buffer before use. Briefly, 1 mL of *S. typhimurium* 01E was mixed with 100 mL of LB broth prepared with the cotton-filtered sewage samples and cotton-filtered supernatant of yak faeces samples. The mixture was overnight cultured at 37°C shaker and then centrifuged (10,000 × *g* for 10 min) and the supernatant was filtered through 0.22 μm pore size filter. Phages in the filtrate were screened by the spot assay and the double-layer agar method (Chang et al., 2005; Gu et al., 2010). Firstly, the spot assay was performed to screen the phage, 100 μL of *S. typhimurium* 01E was spread on 1.5% LB agar plates to prepare the lawns and 10 μL of filtrate was dropped on the lawns and incubated at 37°C for 12 h. The phage was purified by the double layer agar method, the filtrate was mixed with *S. typhimurium* 01E in equal volume, and the mixture was added to 0.75% LB top agar and poured onto 1.5% LB agar plates, then the plates were incubated at 37°C for 12 h. A single plaque was picked up and purified by the double-layer agar method for three times. The purified phage was stored at 4 or −80°C (mixed with 30% glycerol).

Phage morphology was observed as previously described (Kutateladze and Adamia, 2008). Briefly, the phage was amplified (800 mL) and centrifuged (8,000 × *g*, 10 min, 4°C) to remove bacterial debris. Ten percent polyethylene glycol 8,000 and 1 M NaCl were added to the supernatant, incubated in an ice bath overnight and then centrifuged (12,000 × *g*, 20 min, 4°C). The supernatant was removed, and the phage precipitate was resuspended in 2 mL of SM buffer [50 mM Tris HCl, pH 7.5, 0.1 M NaCl, 8 mM MgSO_4_, 0.01% (wt/vol) gelatine]. The concentrated phage was extracted with chloroform. Then, 15 μL of concentrated phage sample was dropped onto 200-mesh copper grids and negatively stained with phosphotungstic acid (2%, w/v). Transmission electron microscopy with an accelerating voltage of 80 kV (HEOL JEM-1200EXII; Japan Electronics and Optics Laboratory, Tokyo, Japan) was used to observe the phage morphology.

The host range of the phage was determined by the double- layer agar plate method (Gu et al., 2010), and the relative efficiency of plating (EOP) was determined, which was expressed as the ratio between the phage titre of the tested strains and the titre of the propagating host strain (Kutter, 2009). All the bacterial strains used to determine the host range of the phages are listed in Supplementary Table 1.

To analyse the thermal stability of phage, phage was incubated at different temperatures (30, 40, 50, 60, 70, and 80°C) for 40 or 80 min. The titre of the phage in the samples before and after incubation was detected by the double-layer agar plate method (Gu et al., 2010).

To analyse the pH stability of the phage, 100 μL of phage was added to 900 μL of SM buffer, which was adjusted to different pH values (2, 4, 6, 8, 10, 12, and 13) and incubated at 37°C for 1 h. The titre of the phage in the samples before and after incubation was detected by the double-layer agar plate method (Gu et al., 2010).

The phage one-step growth curve was determined as previously described (Spellberg et al., 2012). The bacteria (10^8^ CFU/mL) were mixed with phage (10^7^ PFU/mL) for incubating for 10 min, and the mixture was centrifuged (8,500 × *g*, 5 min, 4°C). Then, the precipitate was resuspended in 10 mL of LB broth and cultured at 37°C with shaking at 165 rpm. The 200 μL sample was collected at different time points, and the phage titre in the samples was determined by the double-layer agar plate method (Gu et al., 2010). Burst size was calculated as the ratio of the final number of progeny phage particles to the number of infected bacterial cells (Sritha and Bhat, 2018).

### Phage Genome Sequencing and Analysis

The phage genome was extracted by a virus genome extraction kit (Omega B IO-Tek Inc., Doraville, GA, United States). The whole phage genome was sequenced using the Illumina HiSeq2500 platform at Wuhan GENEWIZ Biotechnology Co., Ltd. (Szabadosova et al., 2018). Genes and predicted open reading frames (ORFs) were assembled using Spades version 3.6.2 with 1,000 times coverage and Gene Marks version 3.6.2, respectively (Besemer et al., 2001; Bankevich et al., 2012). The presence of tRNA was detected by a transcanner.^1^ CLC Genomics Workbench 8.1 (CLC Bio-Qiagen, Aarhus, Denmark) was used to visualise the functional models of the genome (Kim et al., 2013). MEGA version 7.0.26 (Hall, 2013) was used to analyse the evolutionary relationship between isolated phages and reported phages based on the amino acid sequence of the terminal enzyme large subunits (Casjens et al., 2005).

### Bactericidal Effect of Phages *in vitro*

Pc was established by mixing vB_SenS-EnJE1 (NCBI no. MN336264) and vB_SenS-EnJE6 (NCBI no. MN336265) at a ratio of 1:1, and the titre of each phage was 10^9^ PFU/mL. The bactericidal abilities of a single phage and Pc *in vitro* were analysed as previously described (Pajunen et al., 2000).

### The Stability of Phages in the Mouse Gastrointestinal Tract

The stability of phages in the mouse gastrointestinal tract was determined *in vitro* as previously described (Bosak et al., 2018). Three mice were euthanised, and the contents of the stomach, ileum, cecum and colon of each mouse were collected, homogenised and centrifuged (14,000 × *g*, 1 min, 4°C). The supernatant was mixed with the phage (volume 1:1), and the mixtures were incubated at 37°C. Samples were collected at 10, 30, 60, and 90 min after incubation. The phage titre in the samples was determined by the double-layer agar plate method (Gu et al., 2010).

### Animal Experiment

*Salmonella typhimurium* 01E was used to establish the mouse colitis model (Stecher et al., 2006). Briefly, mice were orally administered 20 mg of streptomycin (Hopebio, China) after fasting for 4 h. Then, all mice were supplied with water immediately, and food was supplied *ad libitum* 2 h later. After streptomycin treatment for 20 h, the mice were fasted for 4 h and then orally administered 0.2 mL of *S. typhimurium* 01E (4 × 10^8^ CFU/mouse).

To evaluate the therapeutic effect of the phages, 0.2 mL of 2 × 10^8^ PFU/mouse of a single phage or Pc was orally administered to the mice at 3 h after *S.* Typhimurium infection. As a control, PBS was orally administered to the mouse colitis model.

FMT treatment was performed as previously described (Jacobson et al., 2018). Briefly, fresh faecal pellets were collected from healthy donor mice and homogenised in PBS (2 pellets/mL). Then, the faecal suspensions were immediately centrifuged (500 × *g*, 5 min, 4°C). The supernatants (200 μL/mouse) were gavaged for the first time to mice at 6 h after *S.* Typhimurium infection, and then, the mice continued to receive FMT at 12, 24, and 36 h after *S.* Typhimurium infection.

To evaluate the therapeutic effect of the combination of Pc and FMT (abbreviated as PcFMT), FMT was performed for the first time at 3 h after a single dose of Pc (0.2 mL, 2 × 10^8^ PFU/mouse, at 3 h after infection), and then, the mice continued to receive FMT at 12, 24, and 36 h after *S.* Typhimurium infection. Mice in the control group were gavaged with an equal volume of PBS to replace FMT.

### Disease Activity Index (DAI)

The disease activity index (DAI) of the mice was scored for seven consecutive days using three parameters, including body weight loss, stool consistency and rectal bleeding, as previously described (Zhang et al., 2015). The DAI of different treatment groups was monitored for seven consecutive days. (i) Body weight loss (0, none; 1, 1–5%; 2, 5–10%; 3, 10–15%; 4, >15%), (ii) stool consistency (0, normal; 1, pasty and not adhering to the anus; 2, semiformed adhered to the anus; 3, pasty and adhered to the anus; 4, watery), and (iii) rectal bleeding (0, no blood (–); 1, blood (±); 2, blood (+); 3, blood (++); 4, obvious blood in stool).

### Histopathological Analysis

The colonic tissues of the mice in different treatment groups were collected 48 h after *S.* Typhimurium infection, fixed in 4% paraformaldehyde for 48 h, and then embedded in paraffin for next-step sectioning and H&E (haematoxylin and eosin) staining. The histological score was determined by a histopathological scoring scheme as previously described (Stecher et al., 2006).

### Bacterial Loads in the Colon

At indicated times after infection, mice from different groups were sacrificed. The colonic tissues of the mice were removed. The length of the colon was measured, and the colonic tissues were homogenised in 1 mL of PBS buffer. The bacterial loads of *S.* Typhimurium were determined by plating on *SS* agar plates, and the phage titres were detected by the double-layer agar plate method. The limit of detection (LOD) of bacteria was 10 CFU per organ.

### Enzyme-Linked Immunosorbent Assay

The colonic tissues of mice in different treatment groups were collected 48 h after *S. typhimurium* infection, homogenised in 1 mL of PBS buffer, and centrifuged (10,000 × *g* for 10 min, 4°C). The levels of TNF-α, IL-1β, IL-6, the MPO, and EPO in the supernatant of the colon tissues and the IgA in the serum were measured using Enzyme-linked immunosorbent assay (ELISA) kits (Biolegend, United States) (Strath et al., 1985; Souza et al., 2002; Zhang et al., 2020).

### RT-PCR

Total RNA of colonic tissues was extracted by TRIzol reagent (Invitrogen, United States) and reverse transcribed to cDNA using a PrimeScript RT reagent Kit (Takara, China). The RT-PCR for each gene was repeated in triplicate using SYBR Premix Ex Taq II (Takara, China). The relative expression of SCFA uptake transporters *SMCT*1 and *MCT*1, SCFA signalling genes *HDAC* and *GPR*43, intestine physical barrier-involved genes *Mucin* 1 and *Mucin* 2, and epithelial tight junction related genes (*Occludin*, *Claudin-*3 and *ZO-*1) were calculated by the 2^–ΔΔ*CT*^ method (Livak and Schmittgen, 2001) and normalised according to the expression of β*-actin* and *GAPDH*. The primer sequences used in RT-PCR experiment are listed in Supplementary Table 2.

### Intestinal Microbiota

Fresh faece from each sample was collected and frozen in liquid nitrogen immediately and then stored at −80°C. The total DNA of all samples was extracted using E.Z.N. ATM Mag-Bind Soil DNA Kit (OMEGA, United Kingdom). The 16SV3-V4 region in the 16S rRNA gene was amplified using specific primers (Nobar_341F-805R). Paired-end (PE) reads (250 bp) were generated using the Illumina Hiseq2500 platform. Then, PE reads were merged using PEAR 0.9.8. High-quality clean tags were obtained and clustered into distinct operational taxonomic units (OTUs) by Usearch software with 97% sequence identity. OTUs were further analysed using the RDP classifier against a curated database derived from the RDP database. Alpha diversity, UniFrac beta diversity and principal coordinate analysis (PCoA) were calculated using QIIME. OTUs were also used for determining the differences in taxonomic abundance with group_significance.py.

### Short-Chain Fatty Acids

The supernatants of fresh faeces were collected (10,000 × *g*, 15 min), immediately frozen and stored at −80°C. The concentrations of SCFA, including acetate, butyrate, and propionate, were measured by a gas chromatographic system (Agilent 7890A/5975, United States).

### Statistical Analysis

Statistical analyses of experimental data were performed by SPSS version 13.0 (SPSS, Inc., Chicago, IL, United States), analysed using one-way analysis of variance (ANOVA) and expressed as the means ± SEM. *P* < 0.05 was considered statistically significant. ^∗^*P* < 0.05; ^∗∗^*P* < 0.01; and ^∗∗∗^*P* < 0.001.

## Results

### Characteristics of the Phages

**FIGURE 1:**
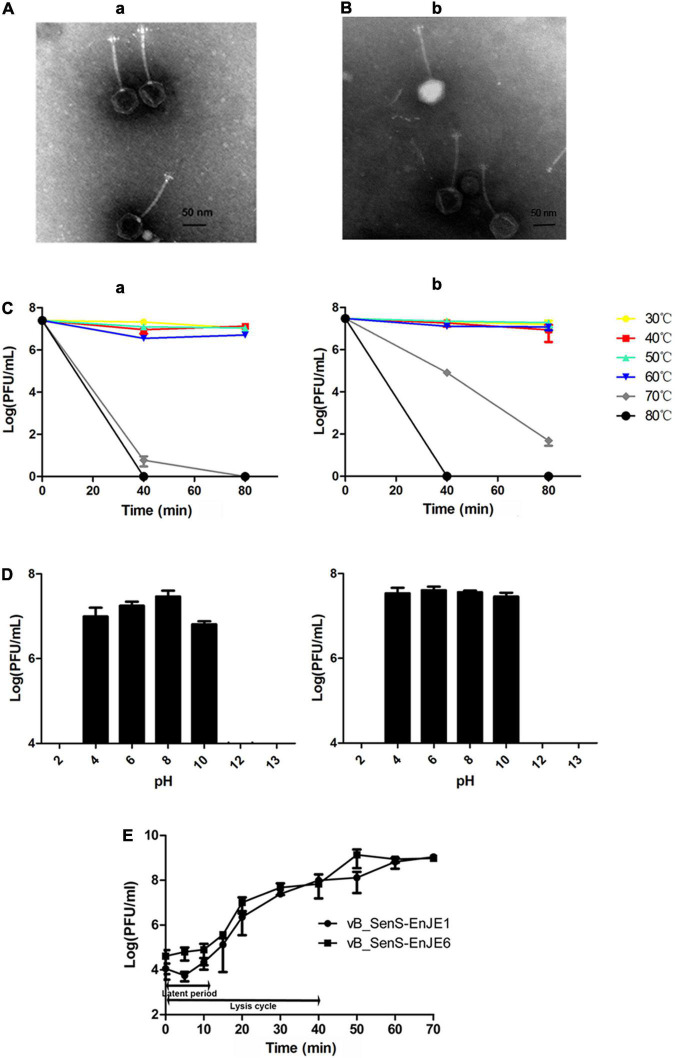
Biological features of phages. Electron microscopy images of vB_SenS-EnJE1 **(A)** and vB_SenS-EnJE6 **(B)**. The bar represents 50 nm. **(C)** Thermal stability of vB_SenS-EnJE1 (a) and vB_SenS-EnJE6 (b). Phage particles were incubated under different temperature conditions, and then, the samples were collected at 40 and 80 min after incubation. **(D)** pH stability of vB_SenS-EnJE1 (a) and vB_SenS-EnJE6 (b). Phage particles were collected after 1 h of incubation under different pH conditions. The titres of the phages at different pH values and thermal conditions were determined by the double layer agar method. **(E)** One-step curves of the two phages at MOI = 0.1. All experiments were repeated three times.

In this study, two *S.* Typhimurium phages were isolated and named vB_SenS-EnJE1 (NCBI no. MN336264) and vB_SenS-EnJE6 (NCBI no. MN336265). TEM showed that vB_SenS-EnJE1 and vB_SenS-EnJE6 belong to the Siphoviridae family, which consists of an icosahedral head with a diameter of approximately 60 ± 5 nm, 60.5 ± 5 and a non-contractile tail with a length of 115 ± 5 nm, 114 ± 5, respectively (Figures 1A,B). The phage titres of vB_SenS-EnJE1 and vB_SenS-EnJE6 changed little from 30 to 60°C (Figure 1C) and pH 4-10 (Figure 1D), indicating that both of them have good temperature and pH stability. vB_SenS-EnJE6 showed a better tolerance to high temperature (70°C) and extreme pH conditions (pH 10) than vB_SenS-EnJE1 (Figures 1C,D). The one-step curves of vB_SenS-EnJE1 and vB_SenS-EnJE6 showed similar latent periods (10 min) and lysis cycles (40 min). In vB_SenS-EnJE1, there was a 5-min eclipse period during which fewer phages could be detected (Figure 1E). The burst sizes of vB_SenS-EnJE1 and vB_SenS-EnJE6 were 88 PFU/cell and 97 PFU/cell, respectively (Figure 1E).

### Host Range

The double layer agar results (Supplementary Table 1) showed vB_SenS-EnJE1 lysis 41/54 strains of *Salmonella*, all of which belong to the O4 serotype. vB_SenS-EnJE6 showed lytic activity against 46/54 *Salmonella* strains, which included the O4 and O9 serotypes (associated with most foodborne illnesses). There were 4 strains (*S. typhimurium*181003, 1811004, 1811007, and *S. dublin SD*) that only can be lysed by vB_SenS-EnJE6. Additionally, vB_SenS-EnJE1 can lyse *Salmonella* strains DE3, Yuch1, Yuch3 and 202035 more efficiently (EOP were higher) than vB_SenS-EnJE6. Otherwise, vB_SenS-EnJE6 showed stronger lytic activity (EOP were higher) than vB_SenS-EnJE1 in lysing strains wudi2, 0078, 01D3, 181008, 181011, and 181017. Additionally, neither vB_SenS-EnJE1 nor vB_SenS-EnJE6 has lysis activity against other species, which showed their high specificity for *Salmonella* species.

### Genomic Characteristics

**FIGURE 2:**
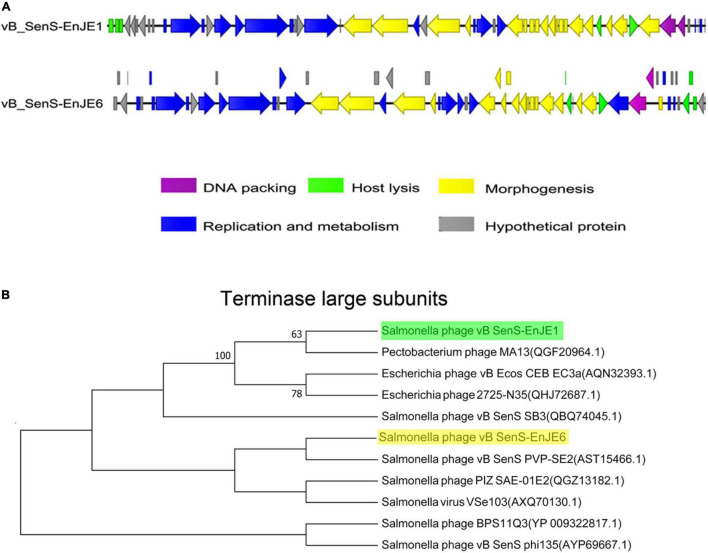
Genome characterisation of phages. **(A)** Comparison analysis of the genomic organisation of vB_SenS-EnJE1 and vB_SenS-EnJE6. CLC workbench 8.1 was employed to visualise the putative CDSs and their transcript direction. The different colours represent different functional clusters. **(B)** Neighbour-joining tree of vB_SenS-EnJE1 and vB_SenS-EnJE6. The evolutionary relationship between isolated phages and reported phages was analysed based on the amino acid sequence of the terminal enzyme large subunits.

vB_SenS-EnJE1 is a linear double-stranded DNA virus composed of 42 894 bp with a GC content of 49.76% (Supplementary Table 3 and Figure 2A). A total of 54 ORFs were predicted, 25.92% were annotated as hypothetical proteins, and one of them showed no significant similarity. vB_SenS-EnJE6 is also a linear double-stranded DNA virus that is 43 129 bp in length and had an average GC content of 49.57%. Its genome carries 69 ORFs, 31.88% of which were annotated as hypothetical proteins (Supplementary Table 4 and Figure 2A). The ORFs of the two phages are shown in Supplementary Tables 3, 4. No lysogen-related genes, virulence factor genes or antibiotic resistance genes were found in vB_SenS-EnJE1 or vB_SenS-EnJE6.

BLAST analysis of their genome against the NCBI on the redundant DNA database was performed. The genome of vB_SenS-EnJE1 was about 94% homogenous to three *Salmonella* phages BPS11Q3 (Accession number: KX405002.1), BPS11T2 (Accession number: MG646668.1), and vB_SpuS_Sp4 (Accession number: MH358359.1) and a *Xanthomonas* phage f29-Xaj (Accession number: KU595434). The vB_SenS-EnJE6 genome showed 94.60% similarity with *Salmonella* SE2 (Accession number: NC_016763.1), about 93% similarity to vB_SenS-Ent1, 2 (Accession number: HE775250.1 and HG934469.1), *Salmonella* phage wksl3 (Accession number: JX202565.1), BPS11Q3 and BPS11T2. Additionally, the nucleotide sequences of vB_SenS-EnJE1 and vB_SenS-EnJE6 showed 95% identity (coverage of 92%) with each other.

Phylogenetic tree analysis showed that vB_SenS-EnJE1 and vB_SenS-EnJE6 belong to the sister clade of reported *Pectobacterium* phage M13 and *Salmonella* phage vB_SenS PVP-SE2, respectively. However, they are at different levels of development (Figure 2B).

### The *in vitro* Bactericidal Effect of the Single Phage and Pc

**FIGURE 3:**
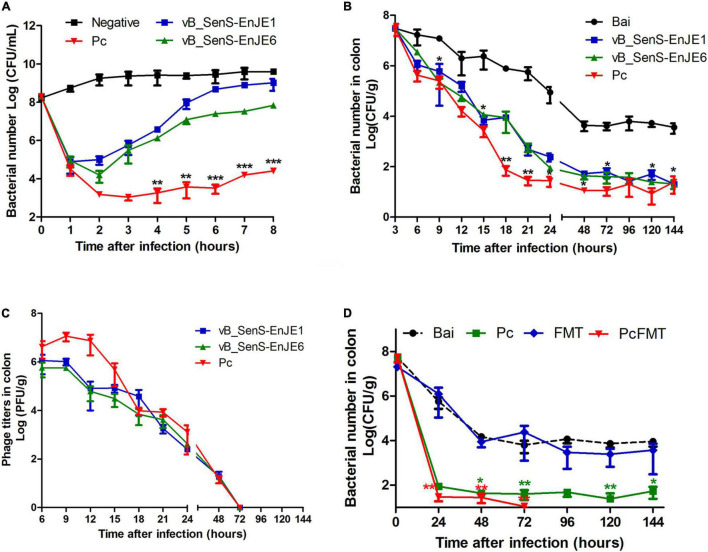
Pc combined with FMT completely removed *S.* Typhimurium *in vivo.* **(A)** The antibacterial effect of single phage and Pc on *S.* Typhimurium *in vitro* at MOI = 0.1. Each independent experiment was repeated three times. The bacterial loads **(B)** and phage titres **(C)** in the colonic tissues of healthy, bacterially infected, single phage-treated and Pc-treated mice. **(D)** The bacterial loads in the colonic tissues of the bacterial-infected, single Pc-treated, and PcFMT-treated mice. Data are expressed as the means ± SEM, n = 6. *P < 0.05; **P < 0.01; ***P < 0.001.

Before the application of the single phage and Pc *in vivo*, their antibacterial activity against *S.* Typhimurium *in vitro* was determined. From Figure 3A, the number of bacteria in the negative control group increased slowly with time from 10^8^ CFU/mL to 10^10^ CFU/mL. The number of bacteria in the single phage treatment group decreased rapidly in the first 1h (vB_SenS-EnJE1) or 2 h (vB_SenS-EnJE6) and then increased rapidly (reaching ≥ 10^8^ CFU/mL at 8 h after treatment). The number of bacteria in the Pc treatment group decreased rapidly (approximately as low as 10^3^ CFU/mL) in the first 2 h and then increased slowly (reaching approximately 10^4^ CFU/mL at 8 h). This shows that Pc can kill *Salmonella* more effectively and delay the emergence of resistant bacteria.

### Therapeutic Effect of the Pc *in vivo*

First, the stability of the single phage and Pc under gastrointestinal conditions was detected. We found that the phage titres were not significantly changed after the single phage or Pc was incubated with the contents of the stomach, ileum, caecum and colon at 37°C for 90 min (Supplementary Figure 1), which showed good stability under gastrointestinal conditions.

**FIGURE 4:**
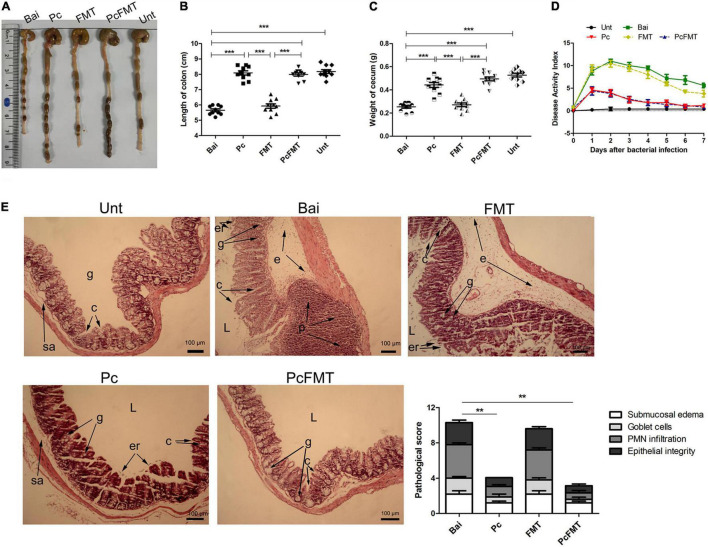
PcFMT treatment effectively improved mouse colitis caused by *S. typhimurium*. The colonic tissues were collected from healthy, bacterial-infected, single-dose Pc-treated, FMT-treated, and PcFMT-treated mice 48 h after *S.* Typhimurium (0.2 mL, 4 × 10^8^ CFU/mouse) infection. “Unt” represents untreated healthy mice group; “Bai” represents the bacterial-infected mice group; “Pc” means phage cocktail-treated mice group; “PcFMT” represents the combined phage cocktail and FMT mice group. **(A)** Macroscopic pathological changes in the colon and caecum. The length of the colon **(B)** and the weight of the caecum **(C)** (n = 6) from untreated, bacterially infected, single-dose Pc-treated, FMT-treated, and PcFMT-treated mice were measured at 48 h post-infection. **(D)** Disease activity index (DAI). **(E)** H&E staining. Colonic tissue was stained with H&E (100×). L, intestinal lumen; e, edema; p, PMN; er, erosion of the epithelial layer; c, crypt; g, goblet cell; sa, submucosa. Magnifications are indicated by the black bars. Data are expressed as the means ± SEM, n = 6. **P < 0.01; ***P < 0.001.

**FIGURE 5:**
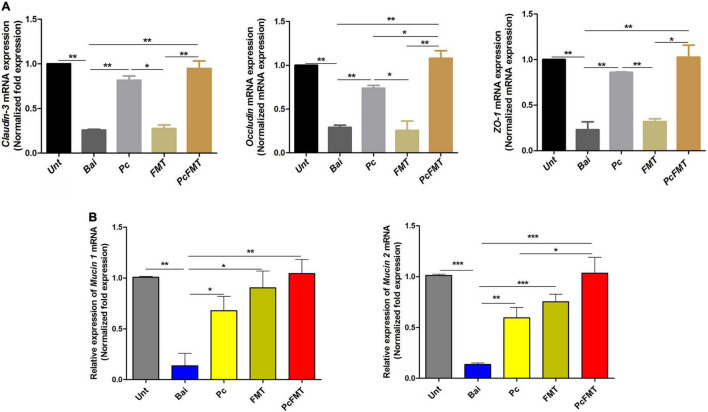
Therapeutic effect of PcFMT on mouse colitis caused by *S.* Typhimurium. The colonic tissues were collected from healthy, bacterial-infected, single-dose Pc-treated, FMT-treated, and PcFMT-treated mice 48 h after *S.* Typhimurium (0.2 mL, 4 × 10^8^ CFU/mouse) infection. “Unt” represents untreated healthy mice group; “Bai” represents the bacterial-infected mice group; “Pc” means phage cocktail-treated mice group; “PcFMT” represents the combined phage cocktail and FMT mice group. **(A)** The transcription levels of the tight junction proteins *Occludin*, *Claudin-*3 and *ZO*-1. **(B)** The transcription levels of epithelial mucus *Mucin* 1 and *Mucin* 2 in colonic tissues. Data are the means ± SEM, n = 6. *P < 0.05; **P < 0.01; ***P < 0.001 indicate a significant difference.

The mouse colitis model was successfully obtained when *S. typhimurium* 01E (0.2 mL, 4 × 10^8^ CFU/mouse) was administered by gavage to the mice. In this model, the length of the colon was significantly shortened (Figures 4A,B). The weight of the caecum was significantly decreased (Figure 4C). The DAI score was increased (Figure 4D). At 24 h after *S.* Typhimurium infection, the bacterial load in the colonic tissues of non-treated mice was approximately 10^4^ CFU/g. The Pc treatment group more effectively reduced the *Salmonella* load (<10^2^ CFU/g) of the mouse colon than a single phage (*P* < 0.05). However, the *Salmonella* remained detectable in colonic tissues of single phage- or Pc-treated mice for 144 h (Figure 3B). In addition, the phage titre in the colonic tissues of the Pc treatment group showed transient proliferation in the first 3 h after gavage and then decreased gradually (Figure 3C). In the colon tissues of mice in the single phage treatment group, there was no significant proliferation of phages, and the titre of phages began to decrease after 3 h. No phage was detected in either the single phage or Pc treatment group at 72 h after gavage. However, Pc treatment can alleviate the severe pathological damage observed in the colon tissue (Figures 4A,E), and recover the transcription levels of the epithelial tight junction related genes (*Claudin*-3, *Occludin* and *ZO*-1) and epithelial mucus genes (*Mucin* 1 and *Mucin* 2) (Figures 5A,B), and restore the contents of inflammatory cytokines (IL-6, IL-1β and TNF-α), MPO and EPO in colonic tissues, and the IgA in blood (*P* < 0.01) (Supplementary Figure 2). All these results indicated that although Pc showed better bactericidal activity and anti-inflammatory activity *in vivo*, Pc could not completely remove *Salmonella* from the colonic tissues of infected mice.

### Therapeutic Effect of PcFMT on Mouse Colitis Caused by *S.* Typhimurium

**FIGURE 6:**
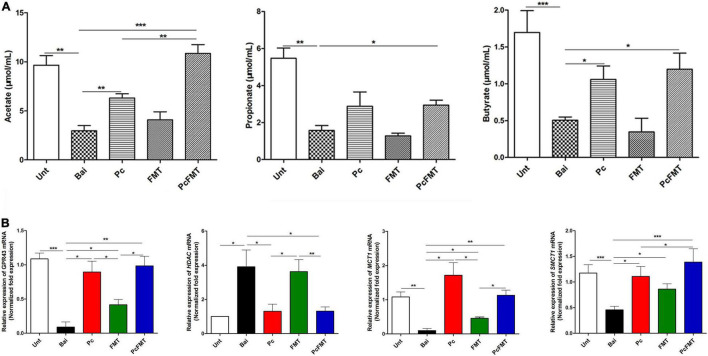
SCFA concentrations and the relative expression of SCFA metabolism related genes. The colonic tissues and faeces were collected from healthy, bacterial-infected, single-dose Pc-treated, FMT-treated, and PcFMT-treated mice 48 h after *S.* Typhimurium (0.2 mL, 4 × 10^8^ CFU/mouse) infection. **(A)** SCFA contents (acetate, propionate, butyrate) in the faeces of healthy, bacteria-infected, single-dose Pc-treated, FMT-treated, and PcFMT-treated mice. **(B)** The transcription levels of the SCFA transporters *SMCT*1 and *MCT*1 and the SCFA receptors *HDAC* and *GPR*43 in the colonic tissues of different treatment groups. The relative expression was calculated as the ratio of the target gene to the internal reference genes (β*-actin* and *GAPDH*). Healthy mice were used as controls. Data are expressed as the means ± SEM, n = 6. *P < 0.05; **P < 0.01; ***P < 0.001.

The therapeutic effect of PcFMT on colitis mice caused by *S.* Typhimurium was further studied. PcFMT completely removed *Salmonella* on the 3rd day (Figure 3D). The length of the colon, the weight of the caecum and DAI score were restored (Figures 4A–D). The pathological damage to the colonic tissues (Figure 4E) and intestinal physical barrier (Figures 5A,B) was repaired. The levels of inflammatory cytokines (IL-6, IL-1β, and TNF-α), MPO and EPO in colonic tissue were significantly reduced. The levels of IgA in blood were also significantly reduced (*P* < 0.05) (Supplementary Figure 2). In addition, compared with the non-treatment group after bacterial infection, the SCFA levels in the faeces of mice in the PcFMT treatment groups were significantly increased, especially the acetate content (*P* < 0.001) (Figure 6A). the transcription level of SCFA metabolism-related genes (*GPR*43, *MCT*1, and *MSCT*1) was also increased (*P* < 0.05). At the same time, the transcription level of the SCFA metabolism gene *HDAC* was significantly decreased (*P* < 0.05) after PcFMT treatment (Figure 6B). Compared with Pc treatment, PcFMT treatment significantly increased the transcription levels of the intestinal barrier-related genes *Occludin* and *Mucin* 2 and the SCFA metabolism-related gene *MSCT*1 (*P* < 0.05) (Figures 5A,B, 6B), and the acetate content also increased (*P* < 0.01) (Figure 6A). Compared with Pc treatment, PcFMT treatment is more conducive to the recovery of the intestinal barrier and the metabolism of SCFA.

### Effect of PcFMT Treatment on Intestinal Microbiota

**FIGURE 7:**
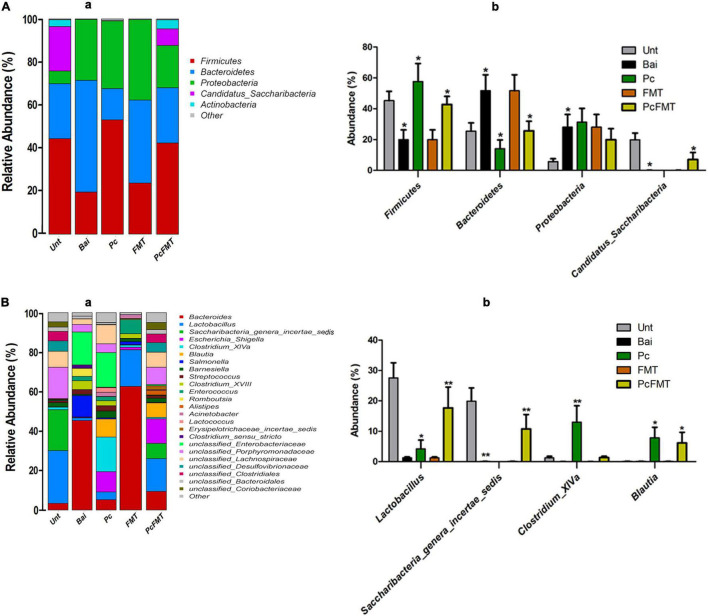
PcFMT recovered the microbiota community structures of *S.* Typhimurium-induced colitis mice. The microbiota of fresh faeces collected from healthy, bacteria-infected, single-dose Pc-treated, FMT-treated, and PcFMT-treated mice were detected by amplifying the V3–V4 region of the 16S rRNA gene using specific primers (Nobar_341F-805R). Relative abundance of predominant bacteria at the phylum **(A)** level and genus **(B)**. “Unt” represents the untreated healthy mice group; “Bai” represents the bacterial-infected mice group; “Pc” means the phage cocktail-treated mice group; “PcFMT” represents the combined phage cocktail and FMT mice group. Data are expressed as the means ± SEM, n = 6. *P < 0.05; **P < 0.01.

To investigate the change of intestinal microbiota after Pc and PcFMT treatment, the species richness and diversity of the intestinal bacterial community of each sample were analysed. Pc or FMT alone had no significant effect on the intestinal microbiota of healthy mice (Supplementary Figure 3). Compared with healthy mice, the intestinal microbiota structure of the mice in the non-treatment group after bacterial infection was significantly destroyed (Supplementary Figures 4A,B). Compared with the non-treatment bacterial infection group, the structure of the intestinal microbiota in the Pc treatment group was partially restored, while that in the PcFMT treatment group was restored to be close to that in the healthy mouse group (Supplementary Figures 4A,B). The results of bacterial phylum classification showed that compared with the intestinal microbiota of healthy mice, the relative abundance of Bacteroidetes and Proteobacteria in the intestinal microbiota of untreated mice after bacterial infection increased significantly (*P* < 0.05), while the relative abundance of *Firmicutes* and *Candidatus_Saccharibacteria* decreased significantly (*P* < 0.05) (Figure 7A). The proportion of Firmicutes and Bacteroidetes in the intestinal tracts of infected mice was partially restored in the Pc treatment group (Figure 7A), while the microbial community structure in the intestinal microbiota of mice in the PcFMT treatment group was restored close to that of healthy mice. At the genus level, the relative abundance of *Lactobacillus* and *Saccharibacteria_genera_incertae_sedis* in the intestinal microbiota of bacterial infection mice without treatment was significantly lower than that in the intestinal microbiota of healthy mice (*P* < 0.01), while PcFMT treatment significantly increased the abundance of *Lactobacillus* (*P* < 0.01) (Figure 7B).

## Discussion

*Salmonella enterica* serovar Typhimurium is a common pathogen that causes invasive intestinal bacterial diseases, such as colitis (Herp et al., 2019). Initially, in this study, we intended to treat the mouse colitis model caused by *S.* Typhimurium using phage therapy. Usually, phage show strong specificity in infecting and killing bacteria. On the one hand, this feature can ensure the safety of phage application without damaging the normal intestinal microbiota of the body. However, at the same time, this feature also limits the application of phage when the antibacterial spectrum is too narrow. Moreover, when only one phage is used, the treated bacteria can quickly produce resistant strains against this phage. Fortunately, Pc can not only maintain specificity for specific pathogens but also broaden the antibacterial spectrum and delay the production of resistant strains (Tanji et al., 2004; Gu et al., 2012; Pires et al., 2020). Therefore, a Pc composed of two phages was used for treatment. In the early stage of treatment, the loads of *S.* Typhimurium in the colon of the colitis model were effectively reduced by Pc, but they could not be completely removed in the later stage. In addition, the analysis of intestinal microbiota showed that Pc treatment had a positive effect on the recovery of intestinal microbiota and reduced the ratio of Bacteroides and Firmicutes, indicating that colitis was alleviated (Wang et al., 2017; Zhang et al., 2018). This may be because most *S.* Typhimurium were killed, which indirectly restored the intestinal microbiota to a certain extent (Hsu et al., 2019). However, the diversity of the intestinal microbiota cannot be completely restored, so *S.* Typhimurium can be colonised for a long time. At the same time, the persistence of *S.* Typhimurium further hinders the recovery of the microbiota (Borewicz et al., 2015).

Antibiotics are the most mainstream treatment therapy for intestinal pathogen infection. However, antibiotics easily cause intestinal microbial disorders (Rivera-Chávez et al., 2016). Moreover, the prevalence of drug-resistant pathogens also greatly reduces the effect of antibiotic therapy (Dodds, 2017). Many studies have shown that FMT is effective in preventing intestinal inflammation caused by chemical substances but not pathogenic bacteria (Wang et al., 2014; Kang et al., 2019). In addition, some case studies have shown that FMT has a good effect in the treatment of human intestinal infections caused by pathogenic bacteria on the premise that antibiotics and other therapies are ineffective (Cohen and Maharshak, 2017). The pathogens in these clinical cases are mainly antibiotic-resistant bacteria, including ESBL-producing and carbapenemase-producing *Enterobacteriaceae*, vancomycin-resistant *Enterococci*, and methicillin-resistant *S. aureus* (Singh et al., 2014; Manges et al., 2016; Davido et al., 2017). Moreover, these bacteria exist in the intestine at a low level, which might be the main reason for the remarkable therapeutic effect (Lahtinen et al., 2017). We used the established mouse colitis model caused by *S.* Typhimurium to evaluate the therapeutic effect of FMT. Unfortunately, the therapeutic effect of FMT on this model was not significant. The loads of *S.* Typhimurium in the colon tissues did not decrease significantly, inflammatory injury was not improved, and the intestinal microbiota did not recover significantly. This may be because FMT was used for treatment rather than prevention in this study, and our treatment time (6, 12, 24, 36 h after infection, observed until 48 h) was shorter than the reported preventive application of FMT (1 time/24 h before infection, at least 1 week) (Jacobson et al., 2018; Hu et al., 2020). In addition, FMT was not able to exert a strong antibacterial effect on *S.* Typhimurium and was not able to effectively reduce the level of inflammation. Inflammation is the main driver of intestinal microbial disorders. Single FMT treatment might only have a transient effect during severe intestinal inflammation and might even produce potential side effects, such as microbial translocation (Winter and Bäumler, 2014; Pigneur and Sokol, 2016).

Could the combination of Pc and FMT have an ideal therapeutic effect? *In vivo* results showed that PcFMT treatment completely eliminated *S.* Typhimurium, significantly alleviated colitis symptoms, restored the intestinal physical barrier, and increased the expression of *Mucin* 1 and *Mucin* 2. Increased expression of *Mucin* 2 has been reported to promote mucus secretion, which contributes to rapid repair of the intestinal mucosa and elimination of foreign pathogens (Birchenough et al., 2015; Mccauley and Guasch, 2015). Additionally, the content of SCFAs, especially acetate after PcFMT treatment were increased significantly. It has been reported that FMT can improve inflammatory diseases caused by bacterial infection by releasing SCFAs (Hu et al., 2020). So, the good treatment effect of PcFMT *in vivo* may be related to SCFAs. Additionally, compared with the bacterial infection group, PcFMT restored the microbial microbiota diversity. It has been reported the balance intestinal microbiota can clear pathogens better (Borewicz et al., 2015). In addition, PcFMT significantly increased the abundance of *Lactobacillus*, *Saccharibateria_genera_incertae_sedis*, and *Blautia*. Especially for the increase in *Lactobacillus*. It has been reported that *Lactobacillus* can effectively reduce the inflammatory level of colitis in mice (Mao et al., 1996). Therefore, the good treatment effect of PcFMT *in vivo* may also be related to the prebiotic effect of increased *Lactobacillus* (Mu et al., 2018).

In conclusion, we found that the combination of Pc and FMT can effectively treat colitis in severe inflammation caused by *S.* Typhimurium. In this combination therapy, phage plays the role of “eliminating pathogen,” that is, specifically killing specific pathogens; FMT mainly plays the role of “strengthening vital qi,” that is, helping to restore the intestinal barrier and microbiota structure and assisting phages in removing *S.* Typhimurium. Because the components of FMT are complex and may even have adverse components (Tan and Johnson, 2019), SCFA or *Lactobacillus* may be directly used to replace FMT and combined with phages of specific pathogens to treat intestinal inflammation caused by corresponding pathogens, and this speculation will be further explored in our follow-up research. This study provides a new idea for the treatment of intestinal inflammatory diseases caused by specific bacterial infections.

## Data Availability Statement

The datasets presented in this study can be found in online repositories. The names of the repository/repositories and accession number(s) can be found in the article/Supplementary Material.

## Ethics Statement

The animal study was reviewed and approved by the Institutional Animal Care and Use Committee (IACUC) of Jilin University.

## Author Contributions

JG and WH conceived the idea and designed the experiment. XW, YX, and YJ carried out the animal experiments. HX and XL performed the bacteriological experiments. XW and YJ analysed the data. LL carried out the bioinformatics analysis. XW and YX wrote the manuscript. JG and WH supervised the study and reviewed the final version of the manuscript. All authors contributed to the article and approved the submitted version.

## Conflict of Interest

The authors declare that the research was conducted in the absence of any commercial or financial relationships that could be construed as a potential conflict of interest.

## Publisher’s Note

All claims expressed in this article are solely those of the authors and do not necessarily represent those of their affiliated organizations, or those of the publisher, the editors and the reviewers. Any product that may be evaluated in this article, or claim that may be made by its manufacturer, is not guaranteed or endorsed by the publisher.

## Footnotes

1
http://lowelab.ucsc.edu/tRNAscan-SE/
