# Supplementary material for: The Combination of Phages and Faecal Microbiota Transplantation Can Effectively Treat Mouse Colitis Caused by Salmonella enterica Serovar Typhimurium
Source: Front Microbiol. 2022 Jul 7;13:944495. doi: 10.3389/fmicb.2022.944495 (PMC9301289; doi:10.3389/fmicb.2022.944495)
Supplement: Supplementary file 1 [file Data_Sheet_1.pdf]

Supplementary Material

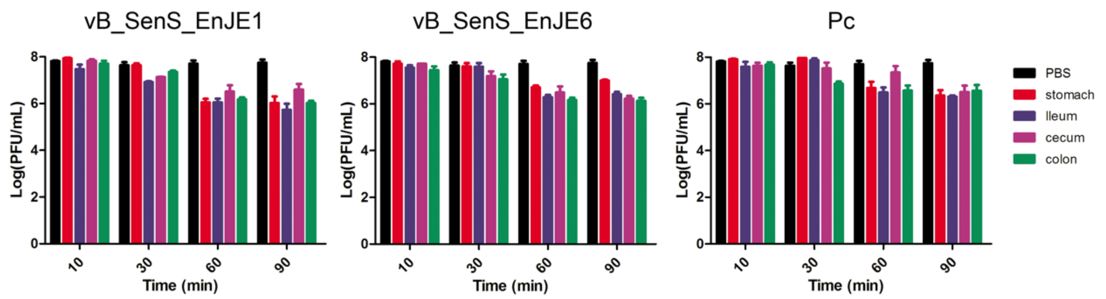

Figure S1. The stability of a single phage and Pc in gastrointestinal conditions. The stability of the phages were measured at 10 min, 30 min, 60 min and 90 min after 1:1 (v/v) coincubation of the phages and contents collected from the stomach, lleum, caecum, and colon of healthy mice at 37 °C. All experiments were repeated three times. Data are the means ± SEM, n = 6. *, P < 0.05; **, P < 0.01; ***, P < 0.001 indicate a significant difference.

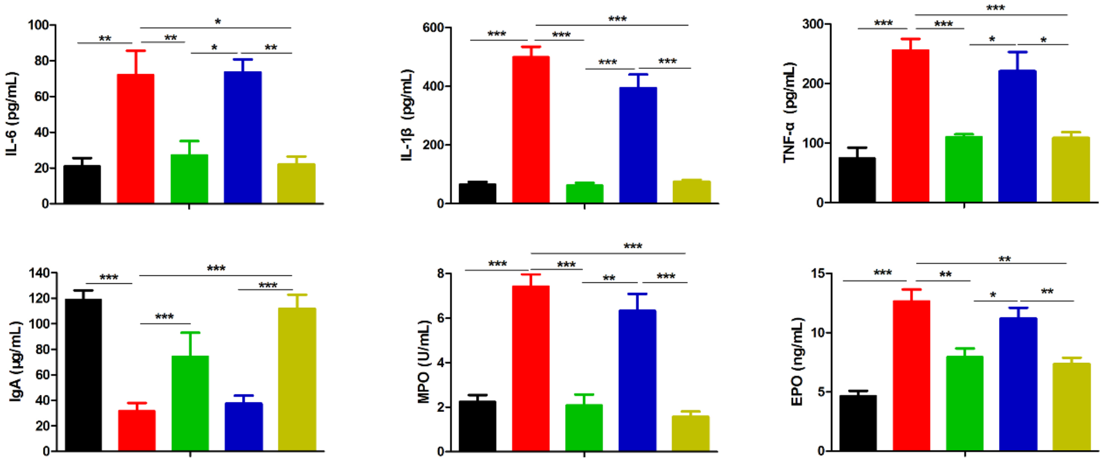

Figure S2. Inflammatory cytokine expression in the colonic tissues. The levels of IL-6, IL-1β, TNF-α, the MPO and EPO in colonic tissues and the IgA in blood were measured. Mice were orally administered with a single dose of Pc (0.2 mL, 2 × 10^8

PFU/mouse) at 3 h after *S. typhimurium* 01E infection) by gavage or with FMT (0.2 mL/mouse) at 6, 12, 24 and 36 h after *S. typhimurium* 01E infection. Healthy mice were used as controls. Data are expressed as the means ± SEM, n = 6. *, $P < 0.05$; **, $P < 0.01$; ***, $P < 0.001$.

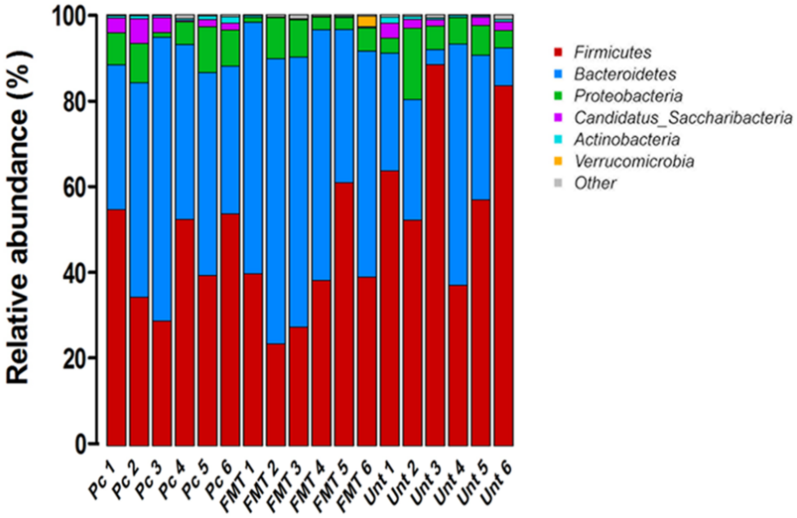

Figure S3. Single dose Pc or single FMT treatment has no side effect on intestinal microbiota of healthy mice. Relative abundance of predominant genera in faeces. Mice were orally administered with a single dose of Pc (0.2 mL, 2 × 10^8 PFU/mouse) or with FMT (0.2 mL/mouse) at different time intervals. The microbiota of fresh faeces collected from healthy, single-dose Pc-treated and FMT-treated mice were detected by amplifying the V3-V4 region of the 16S rRNA gene using specific primers (Nobar_341F-805R).

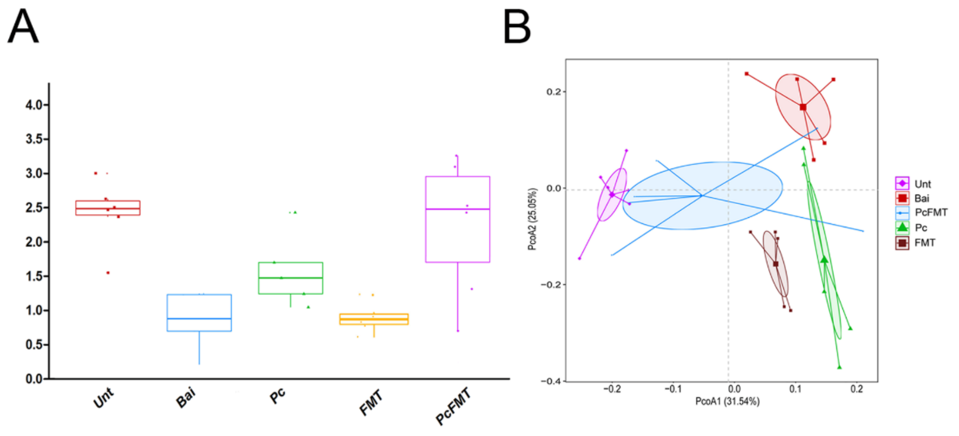

Figure S4. PcFMT recovered the microbiota community structures of *S.* Typhimurium-induced colitis mice. The microbiota of fresh faeces collected from mice of all treatment groups were detected by amplifying the V3-V4 region of the 16S rRNA gene using specific primers (Nobar_341F-805R). (A) Alpha diversity was measured by the Shannon index. (B) PCoA plot of the faecal microbiota was estimated based on an unweighted UniFrac metric. “Unt” represents untreated healthy mice group; “Bai” represents the bacterial-infected mice group; “Pc” means phage cocktail-treated mice group; “PcFMT” represents the combined phage cocktail and FMT mice group.

Table S1. The host range of vB_SenS-EnJE1 and vB_SenS-EnJE6.

Bacterial strain	EOP		Double-layer agar method		Bacterial strain	EOP		Double-layer agar method	
	01E1	01E6	01E1	01E6		01E1	01E6	01E1	01E6
S. Typhimurium 01E [1]	2.15	2.21	++	++	*S.* Abortusequi DE1 [4]	1.22	1.42	++	++
S. Typhimurium ATCC14028 [2]	1.59	1.47	++	++	*S.* Abortusequi DE2 [4]	1.05	0.97	++	++
S. Typhimurium 181001 [3]	1.29	1.41	++	++	*S.* Abortusequi DE3 [4]	1.12	0.48	++	+
S. Typhimurium 181002 [3]	1.81	2.05	++	++	*S.* Abortusequi DE4 [4]	0.89	0.95	++	++
S. Typhimurium 181003 [3]	-	2.41	-	++	*S.* Abortusequi DE5 [4]	0.95	1.07	+	+
S. Typhimurium 181004 [3]	-	2.53	-	++	*S.* Abortusequi DE6 [4]	1.05	0.91	++	++
S. Typhimurium 181005 [3]	1.89	1.76	++	++	*S.* Abortusequi DE7 [4]	0.75	0.87	++	++
S. Typhimurium 181006 [3]	1.59	1.71	++	++	*S.* Abortusequi GT1 [4]	1.65	1.42	++	++
S. Typhimurium 181007 [3]	-		-	++	*S.* Abortusequi GT2 [4]	1.01	1	++	++
S. Typhimurium 181008 [3]	0.59	1.15	+	++	*S.* Abortusequi 0078 [4]	0.42	0.91	+	++
S. Typhimurium 181009 [3]	1.84	1.97	++	++	*S.* Abortusequi 078H [4]	0.98	1.17	++	++
S. Typhimurium 181011 [3]	1.03	2.58	+	++	*S.* Abortusequi 0514 [4]	1.4	1.7	++	++
S. Typhimurium 181012 [3]	1.25	1.48	++	++	*S.* Abortusequi 202035 [4]	1.2	0.58	++	+
S. Typhimurium 181013 [3]	1.51	1.76	++	++	*S.* Abortusequi 23EE [4]	0.9	0.95	++	++
S. Typhimurium 181014 [3]	1.75	1.42	++	++	*S.* Abortusequi 01D3 [4]	0.34	0.78	+	++
S. Typhimurium 181015 [3]	1.38	1.58	++	++	*S.* Dublin SD [1]	-	1.1	-	++
S. Typhimurium 181016 [3]	1.17	1.65	++	++	*S.* Pullorum SP [1]	-	-	-	-
S. Typhimurium 181017 [3]	0.85	2.14	+	++	*S.* Pullorum S06004 (Li *et al*., 2015)	-	-	-	-
S. Typhimurium 181018 [3]	0.97	1.91	++	++	*S.* Abortusequi ATCC9842 [2]	-	-	-	-
S. Typhimurium 181019 [3]	1.51	1.49	++	++	*S.* Enteritidis ATCC13076 [2]	0.8	0.9	++	++
S. Typhimurium LH430 [1]	-	-	-	-	*S.* Livingstone 00750 [1]	-	-	-	-
S. Typhimurium 1344 (Hu *et al*., 2017)	-	-	-	-	*S.* Livingstone 00751 [1]	-	-	-	-
S. Typhimurium 20210101 [1]	2.13	2.34	++	++	*S.* Livingstone 00752 [1]	-	-	-	-
S. Typhimurium 202012 [1]	1.91	2.01	++	++	*Salmonella* 1021 [1]	-	-	-	-
S. Abortusequi wudi1 [4]	1.79	2.13	++	++	*E. coli* 011D [4]	-	-	-	-
S. Abortusequi wudi2 [4]	0.97	2.51	+	++	*E. coli* E1[1]	-	-	-	-
S. Abortusequi Yuch1 [4]	1.54	0.98	++	+	*E. coli* E4 [5]	-	-	-	-
S. Abortusequi Yuch2 [4]	2.11	2.09	++	++	*K. pneumoniae* K7 (Cai *et al*., 2018)	-	-	-	-
S. Abortusequi Yuch3 [4]	1.45	0.67	++	+	*S. aureus* N38 (*Gu et al., 2010*)	-	-	-	-
S. Abortusequi Yuch4 [4]	21.9	2.39	++	++	*E. faecalis* GF29 (Cheng *et al*., 2017)	-	-	-	-

[1] laboratory preserved strains; [2] purchased strains from the China Center of Industrial Culture Collection; [3] were kindly provided by Yuqing Liu (Shandong Academy of Agricultural Sciences, Jinan, China) (strains were isolated from Jinan Chicken Farms); [4] were kindly provided by Jianbao Dong (Liaocheng University, Liaocheng, China) (strains were isolated from Shandong Donkey Farms); [5] were kindly provided by the First Hospital of Jilin Province. (++) indicates transparent plaques were observed; (+) indicates blurred plaques were observed; (-) indicates no plaque was observed.

Table S2. The primers used for the RT–PCR analysis in this study.

Genes	Primer sequence (5' to 3')
*SMCT*1	F: TGCCATTTCCTTATGGGTAGG
	R: AGTGGAGTCCTTTCCGCATTA
*MCT*1	F: GTGCAGCA-GCCAAGGAGCCC
	R: CCATGGCCAGTCCGTTGGCC
*GPR*43	F: CTGTATGGAGTGATGATCGCTGCTCTG
	R: CTGCTCTTGGGTGAAGTTCTCGTAG
HDAC	F: CACACAGCAGACTTTCTACCAGGAC
	R: GACATTGAAGCCCTCGCCACTG
Mucin 1	F: CCTTCAGTGCCAAGTCAATAC
	R: TCCCCAGAAAATCTCCGTT
Mucin 2	F: ATGCCCACCTCCTCAAAGAC
	R: GTAGTTTCCGTTGGAACAGTGAA
β-actin	F: CAGCTGAGAGGGAAATCGTG
	R: CTCCAGGGAGGAAGAGGATG
GAPDH	F: CCATGGAGAAGGCTGGGG
	R: CAAAGTTGTCATGGATGACC

Table S3. General features of the putative ORFs of vB_SenS-EnJE1 with the best matches in the database.

ORFa	Bp Start	Bp Stop	Amio acid	Protein size (kDa)	pI	Annotated function	Representative similarity to proteins in database	E value	Identity (positives)	Query cover	Accession no.
1† -	102	458	118	12.517	9.85	hypothetical protein	[*Salmonella* phage BPS11Q3]	3e-79	100%	100%	YP_009322827.1
2†-	568	858	96	10.648	9.98	putative class I holin	[*Salmonella* phage SETP13]	1e-61	96.88%	100%	YP_008767059.1
3§-	860	1141	93	10.260	7.85	putative holin	[*Salmonella* phage Jersey]	5e-59	94.62%	100%	YP_008239771.1
4† -	1220	1654	144	15.574	4.95	hypothetical protein	[*Salmonella* phage LPSE1]	4e-101	100%	100%	APU02983.1
5† -	1660	2031	123	14.144	10.46	hypothetical protein	[*Salmonella* enterica]	2e-84	99.19%	99%	WP_129416900.1
6‡-	2034	2210	58	6.329	7.04	hypothetical protein	[*Salmonella* phage SE2]	4e-35	100%	100%	YP_005098114.1
7† -	2234	2743	169	19.412	5.13	hypothetical protein	[*Salmonella* phage BPS11Q3]	2e-99	86.47%	100%	YP_009322833.1
8† -	2913	3077	54	6.376	11.82	hypothetical protein	[*Salmonella* phage wksl3]	7e-30	98.15%	100%	AFO12358.1
9† -	3198	3368	56	5.699	9.74	No significant similarity found					
10‡ +	4011	4241	76	8.877	8.00	DNA-binding protein	[*Salmonella* phage BPS11T2]	1e-34	95.08%	80%	AUM59064.1
11†+	4528	6714	728	80.411	4.88	primase/helicase	[*Salmonella* phage vB_SenS_phi135]	0.0	99.18%	100%	AYP69663.1
12† -	6728	6946	72	7.920	8.57	XRE family transcriptional regulator	[*Salmonella enterica*]	1e-45	100%	100%	WP_015984911.1
13† +	7080	7592	170	19.288	4.50	hypothetical protein	[*Salmonella* phage SETP7]	2e-97	90.59%	100%	YP_008767212.1
14† +	7634	8875	413	46.036	7.30	DUF2800 domain-containing protein	[*Salmonella enterica*]	0.0	93.46%	100%	EAA8503712.1
15†+	9135	9764	209	23.630	4.76	DNA polymerase	[*Salmonella* phage BPS11Q3]	2e-149	98.56%	100%	YP_009322844.1
16† +	9822	12920	1032	115.874	7.63	DNA polymerase I	[*Salmonella* phage vB_SpuS_Sp4]	0.0	95.93%	100%	AWY03025.1
17† +	13007	13294	95	10.880	9.63	putative restriction endonuclease	[*Salmonella* phage LPSE1]	1e-61	96.84%	100%	APU03027.1
18† +	13326	13517	63	7.038	9.72	hypothetical protein	[*Salmonella* phage LPSE1]	2e-35	96.83%	100%	APU03026.1
19† +	13519	14016	165	18.829	9.85	hypothetical protein	[*Salmonella* phage STP03]	3e-115	96.97%	100%	APM00325.1
20† +	14049	16478	809	90.800	8.01	putative helicase	[*Salmonella* phage wksl3]	0.0	97.78%	100%	AFO12372.1
21† +	16553	16696	47	5.760	6.67	hypothetical protein	[*Salmonella* phage SETP13]	5e-22	100%	100%	YP_008767032.1
22† -	16815	18869	684	72.931	5.06	tail spike protein	[S *Salmonella* virus VSt10]	0.0	98.39%	100%	AXQ70291.1
23‡ -	18882	21440	852	93.910	5.16	tail fiber protein	[*Salmonella* phage vB_SpuS_Sp4]	0.0	99.30%	100%	AWY03031.1
24† -	21793	22245	150	16.905	4.21	DUF1833 domain-containing protein	[*Salmonella* enterica]	8e-106	99.33%	100%	WP_015976748.1
25† -	22305	22805	166	18.638	4.72	hypothetical protein	[*Salmonella* phage SETP13]	1e-121	100%	100%	YP_008767027.1
26† -	22807	25140	777	82.970	4.66	putative tape measure protein	[*Salmonella* phage LPSE1]	0.0	98.71%	100%	APU03018.1

27† -	25498	25914	138	15.849	5.24	tail assembly chaperone	[*Salmonella* phage vB_SenS_PVP-SE2]	3e-95	98.55%	100%	AST15484.1
28†+	26084	26263	59	6.603	9.63	superinfection immunity protein	[*Salmonella enterica*]	2e-32	100%	100%	WP_015984947.1
29† +	26326	27456	376	42.485	8.82	oxidoreductase	[*Salmonella enterica*]	0.0	99.47%	100%	WP_064624764.1
30†+	27477	27683	68	7.681	5.09	hypothetical protein	[*Salmonella enterica*]	2e-42	100%	100%	WP_015984945.1
31† +	27797	28468	223	25.665	7.30	putative DNA-binding protein	[*Salmonella enterica*]	1e-16	98.65%	100%	WP_016062375.1
32† -	28497	29639	380	40.213	4.35	putative tail protein	[*Salmonella* phage wksl3]	0.0	98.42%	100%	AFO12387.1
33† -	29666	29947	93	10.113	5.11	phage tail protein	[*Salmonella* phage FSL SP-101]	7e-59	94.62%	100%	AGF87719.1
34§ -	30085	30450	121	13.321	9.77	tail protein	[*Salmonella* phage BPS11Q3]	5e-84	100%	100%	YP_009322866.1
35† -	30477	30812	111	12.176	8.37	tail protein	[*Salmonella* phage BPS11Q3]	1e-73	100%	100%	YP_009322867.1
36† -	30836	31441	201	20.566	8.45	neck protein	[*Salmonella* phage BPS11Q3]	2e-140	100%	100%	YP_009322868.1
37† -	31444	31953	169	17.778	4.60	head-tail joining protein	[*Salmonella* phage BPS11Q3]	9e-118	98.22%	100%	YP_009322869.1
38† -	31957	32145	62	7.167	4.85	head-tail joining protein	[*Salmonella* phage STP03]	4e-35	96.77%	100%	APM00274.1
39† -	32182	32532	116	12.244	4.02	neck whiskers protein	[*Salmonella* phage STP03]	5e-75	98.28%	100%	APM00275.1
40† -	32544	32828	94	9.325	9.79	head-tail joining protein	[*Salmonella* phage BPS11Q3]	4e-57	100%	100%	YP_009322872.1
41† -	32889	33938	349	37.856	4.61	coat protein	[*Salmonella* phage BPS11T2]	0.0	)99.14%	100%	AUM59037.1
42† -	33942	34643	233	25.692	6.13	putative scaffold protein	[*Salmonella* phage SETP3]	2e-162	97.85%	94%	YP_001110836.1
43† -	34837	35223	128	14.164	9.47	o-spanin	[*Salmonella* phage vB_SenS_PVP-SE2]	5e-83	94.53%	100%	AST15489.1
44† -	35541	35999	152	16.393	4.43	neck whiskers protein	[*Salmonella* phage vB_SpuS_Sp4]	8e-101	99.34%	100%	AWY02990.1
45† -	36002	37075	357	40.094	6.79	head morphogenesis protein	[*Salmonella* phage FSL SP-101]	0.0	94.60%	98%	AGF87707.1
46† +	37211	37861	216	24.982	5.75	amidase	[*Salmonella* phage SE2]	2e-153	94.44%	100%	YP_005098129.1
47† -	37892	39364	490	53.813	4.68	62kDa structural protein	[*Salmonella* phage S119]	0.0	99.18%	100%	AXC41123.1
48† -	39377	40516	379	43.019	6.27	putative phage terminase large subunit	[*Salmonella* phage SETP7]	0.0	99.47%	100%	YP_008767173.1
49† -	40638	41183	181	20.479	6.27	putative terminase small subunit	[*Salmonella* phage SETP3]	2e-130	99.45%	100%	YP_001110829.1
50† -	41365	41547	60	6.858	8.68	hypothetical protein	[*Salmonella* phage SE2]	2e-34	100%	100%	YP_005098124.1
51† -	41846	41992	48	5.752	10.04	putative NinH-like protein	[*Salmonella* phage Ent1]	1e-26	97.92%	100%	YP_007010509.1
52† -	42067	42177	36	4.284	4.12	hypothetical protein	[*Salmonella* phage vB_SpuS_Sp4]	3e-13	94.44%	100%	AWY03000.1

53† -	42213	42395	60	7.119	6.35	putative NinZ-like protein	[*Salmonella* phage SETP3]	4e-34	96.67%	100%	YP_001110826.1
54† -	42544	42729	61	6.880	7.08	hypothetical protein	[*Salmonella* phage SE2]	8e-38	100%	100%	YP_005098119.1

[a] †, AUG start codons; ‡, UUG start codons; §, GUG start codons. +, right orientation; -, left orientation.

Table S4. General features of the putative ORFs of vB_SenS-EnJE6 with the best matches in the database.

ORF[a]	Bp		Amio acid	Protein size (kDa)	pI	Annotated function	Representative similarity to proteins in database	E value	Identity (positives)	Query cover	Accession no.
	Start	Stop									
1† -	2	277	92	10.287	10.23	hypothetical protein	[*Salmonella enterica*]	1e-60	100%	100%	WP_129416900.1
2†-	280	483	67	7.406	7.04	hypothetical protein	[*Salmonella* phage SE2]	1e-42	100%	100%	YP_005098114.1
3† -	480	995	171	19.628	6.11	hypothetical protein	[*Salmonella* phage vB_SpuS_Sp4]	1e-106	88.69%	98%	AWY03012.1
4† -	988	1101	37	4.319	3.35	hypothetical protein	[*Salmonella* phage BPS11Q3]	8e-19	100%	100%	YP_009322834.1
5† -	1571	1672	33	3.866	9.84	DUF551 domain-containing protein	[*Salmonella enterica*]	3e-12	87.88%	100%	WP_078055194.1
6†-	1669	1944	91	11.030	8.66	ASCH domain-containing protein	[*Salmonella enterica*]	6e-58	94.51%	100%	WP_000002128.1
7† -	1960	2163	67	7.575	9.75	hypothetical protein	[*Salmonella* phage vB_SpuS_Sp4]	4e-42	100%	100%	AWY03016.1
8† +	2621	2791	56	6.636	10	DNA-binding protein	[*Salmonella* phage BPS11T2]	3e-33	100%	100%	AUM59064.1
9†+	2788	3021	77	8.641	9.49	putative uvsX-like protein	[*Salmonella* phage Ent1]	8e-46	96.10%	100%	YP_007010497.1
10†+	3078	5264	728	80.332	4.87	hypothetical protein	[*Salmonella* phage vB_SenS_phi135]	0.0	99.18%	100% %	AYP69663.1
11† -	5279	5497	72	7.920	8.57	XRE family transcriptional regulator	[*Salmonella enterica*]	1e-45	100%	100%	WP_015984911.1
12†+	5631	6143	170	19.245	4.45	hypothetical protein	[*Salmonella* phage SETP7]	2e-98	91.18%	100%	YP_008767212.1
13†+	6185	7426	413	46.006	7.30	DUF2800 domain-containing protein	[*Salmonella* enterica]	0.0	93.70%	100%	EAA8503712.1
14† +	7423	7596	57	6.771	9.69	hypothetical protein	[*Salmonella* phage vB_SpuS_Sp4]	5e-32	100%	100%	AWY03023.1
15† +	7686	8315	209	23.617	4.76	DUF2815 family protein	[*Salmonella enterica*]	2e-149	98.56%	98%	WP_076915462.1
16† +	8373	11477	1034	116.196	7.53	intein-containing DNA polymerase precursor	[*Salmonella* phage BPS11Q3]	0.0	)96.62%	100%	YP_009322845.1
17†+	11564	11851	95	10.880	9.63	hypothetical protein	[*Salmonella* phage LPSE1]	1e-61	96.84%	100%	APU03027.1
18† +	11883	12074	63	7.098	8.54	hypothetical protein	[*Salmonella* phage vB_SpuS_Sp4]	2e-36	98.41%	100%	AWY03027.1
19† +	12071	12574	167	18.916	9.66	HNH endonuclease	[*Salmonella* virus VSiP]	4e-30	39.51%	97%	AXQ70220.1
20†+	12567	13991	474	53.396	8.75	putative helicase	[*Escherichia* phage vB_EcoS-Ro145clw]	0.0	)97.47%	100%	AUX83700.1
21†+	13988	14209	73	8.722	4.75	hypothetical protein	[*Salmonella* phage SETP13]	6e-42	100%	100%	YP_008767032.1
22† +	14206	14298	30	3.069	9.00	hypothetical protein	[*Salmonella* phage SG2]	3e-23	100%	100%	ASZ76550.1
23† -	14328	16382	684	72.756	5.01	tailspike protein	[*Salmonella* phage L13]	0.0	97.22%	100%	YP_008058250.1
24† -	16395	18953	852	93.747	5.31	tail fiber protein	[*Salmonella* phage BPS11T2]	0.0	99.06%	100%	AUM59079.1

25† -	18944	19309	121	13.911	6.60	hypothetical protein	[*Salmonella* phage vB_SpuS_Sp4]	5e-86	99.17%	100%	AWY03032.1
26§-	19306	19821	171	19.197	4.29	DUF1833 domain-containing protein	[*Salmonella enterica*]	8e-122	98.83%	100%	WP_015976748.1
27† -	19818	20318	166	18.652	4.76	hypothetical protein	[*Salmonella* phage SETP13]	3e-121	99.40%	100%	YP_008767027.1
28† -	20320	22653	777	83.017	4.74	tail tape measure protein	[*Salmonella* virus VSt10]	0.0	97.68%	100%	AXQ70286.1
29† -	22646	23005	119	13.581	4.55	hypothetical protein	[*Salmonella* phage SE2]	2e-83	100%	100%	YP_005098151.1
30† -	23011	23427	138	15.847	4.96	tail assembly chaperone	[*Salmonella* phage vB_SenS_PVP-SE2]	6e-96	99.28%	100%	AST15484.1
31†+	23597	23776	59	6.498	9.63	superinfection immunity protein	[*Salmonella enterica*]	2e-3	94.92%	100%	WP_015984947.1
32†+	23839	24993	384	43.464	8.49	putative serine/theonine protein phosphatase	[*Salmonella* phage vB_SenS-Ent2]	0.0	97.41%	100%	YP_009009947.1
33†+	25009	25509	166	19.285	9.93	putative HNH endonuclease	[*Salmonella* phage wksl3]	1e-118	100%	100%	AFO12384.1
34† +	25519	25749	76	8.690	6.33	hypothetical protein	[*Salmonella enterica*]	7e-49	100%	100%	WP_015984945.1
35†+	25862	26533	223	25.665	7.30	putative DNA-binding protein	[*Salmonella enterica*]	1e-161	98.65%	100%	WP_016062375.1
36§-	26562	27731	389	41.068	4.49	hypothetical protein	[*Salmonella* phage vB_SenS-Ent2]	0.0	97.69%	100%	YP_009009943.1
37 ‡ -	27731	28150	139	15.137	4.22	phage tail protein	[*Salmonella* phage FSL SP-101]	3e-88	90.65%	100%	AGF87719.1
38†-	28150	28545	131	14.430	9.71	HK97 gp10 family phage protein	[*Salmonella* enterica]	1e-90	98.47%	100%	WP_015984941.1
39†-	28542	28901	119	13.115	9.43	tail protein	[*Salmonella* phage BPS11Q3]	1e-77	96.64%	100%	YP_009322867.1
40† -	28901	29506	201	20.648	9.10	Putative neck protein	[*Salmonella* phage Ent1]	1e-136	98.01%	94%	YP_007010468.1
41† -	29509	30018	169	17.734	4.60	putative head-tail joining protein	[*Salmonella* phage vB_SenS-Ent2]	5e-116	97.04%	100%	YP_009009938.1
42† -	30022	30210	62	7.196	5.07	putative head-tail joining protein	[*Salmonella* phage STP03]	2e-34	95.16%	100%	APM00274.1
43† -	30247	30597	116	12.225	4.31	neck whiskers protein	[*Salmonella* phage S100]	2e-75	98.28%	100%	AXC39494.1
44† -	30609	30896	95	9.425	9.54	putative head protein	[*Salmonella* phage SE2]	2e-58	100%	100%	YP_005098136.1
45† -	30957	32006	349	37.926	4.62	putative major coat protein	[*Salmonella* phage Ent1]	0.0	98.85%	100%	YP_007010463.1
46† -	32010	32711	233	25.780	6.43	scaffold protein	[*Salmonella* virus VSt10]	1e-162	98.71%	100%	AXQ70268.1
47† -	32796	32918	40	4.693	4.30	scaffold protein	[*Salmonella* phage Jersey]	4e-19	97.50%	100%	YP_008239719.1
48† -	32905	33291	128	14. 247	9.45	o-spanin	[*Salmonella* phage vB_SenS_PVP-SE2]	4e-77	89.06%	100%	AST15489.1
49† -	33336	33455	39	4.653	10.98	hypothetical protein	[*Salmonella* phage ST3]	3e-20	100%	100%	ASZ77969.1
50† -	33609	34067	152	16.403	4.39	putative head decoration protein	[*Salmonella* phage Ent1]	5e-100	98.03%	100%	YP_007010459.1

51‡ -	34070	35113	347	38.811	5.47	putative head morphogenesis protein, SPP1 gp7 family	[*Salmonella* phage vB_SenS-Ent2]	0.0	96.54%	100%	YP_009009929.1
52† +	35279	35929	216	24.966	5.75	amidase	[*Salmonella* phage SE2]	1e-153	94.91%	100%	YP_005098129.1
53† -	35960	37432	490	53.844	4.68	DUF4055 domain-containing protein	[*Salmonella* virus VSe103]	0.0	99.39%	100%	AXQ70131.1
54†-	37445	38716	423	47.568	6.35	terminase large subunit	[*Salmonella* phage vB_SenS_SB3]	0.0	99.76%	100%	CRI74278.1
55† -	38706	39251	181	20.435	6.23	putative terminase small subunit	[*Salmonella* phage SETP3]	2e-129	98.34%	100%	YP_001110829.1
56† -	39335	39436	33	3.705	10.08	hypothetical protein	[*Citrobacter* virus Stevie]	7e-09	63.64%	100%	YP_009148759.1
57† -	39433	39615	60	6.858	8.68	hypothetical protein	[*Salmonella* phage SE2]	2e-34	100%	100%	YP_005098124.1
58†-	39612	39914	100	12.181	9.67	putative membrane protein	[*Salmonella* phage vB_SenS_PVP-SE2]	6e-42	79.12%	91%	AST15499.1
59† -	39914	40138	74	8.578	9.27	putative NinH-like protein	[*Salmonella* phage Ent1]	2e-46	97.30%	100%	YP_007010509.1
60† -	40281	40511	76	8.855	6.58	putative NinZ-like protein	[*Salmonella* phage SETP3]	2e-24	86.54%	68%	YP_001110826.1
61†-	40504	40689	61	7.316	6.59	hypothetical protein	[*Salmonella* phage vB_SpuS_Sp4]	4e-38	100%	100%	AWY03002.1
62†-	40686	40841	51	5.798	10.01	DUF2737 domain-containing protein	[*Salmonella* phage vB_SenS_phi135]	1e-274e-157	94.12%	100%	AYP69714.1
63† -	40838	40996	52	6.060	3.90	hypothetical protein	[*Salmonella* phage vB_SpuS_Sp4]	4e-27	98.08%	97%	AWY03004.1
64† -	40993	41178	61	6.893	8.17	hypothetical protein	[*Salmonella* phage SS3e]	1e-37	100%	100%	YP_224044.1
65† -	41362	41850	162	17.341	9.66	lysin	[*Salmonella* phage vB_SpuS_Sp4]	2e-112	97.53%	100%	AWY03006.1
66†-	41828	42118	96	10.693	9.98	putative holin, class II	[*Salmonella* phage vB_SenS-Ent2]	8e-62	97.89%	98%	YP_009009974.1
67†-	42120	42401	93	10.321	7.85	putative holin	[*Salmonella* phage Jersey]	2e-59	94.62%	100%	YP_008239771.1
68†-	42480	42914	144	15.397	4.46	hypothetical protein	[*Salmonella enterica*]	6e-97	95.83%	100%	WP_129416902.1
69†-	42920	43129	69	8.098	10.53	hypothetical protein	[*Salmonella* phage BPS11Q3]	3e-44	100%	100%	YP_009322831.1

[a] †, AUG start codons; ‡, UUG start codons; §, GUG start codons. +, right orientation; -, left orientation.
